# Initial Medication in Patients of Newly Diagnosed Parkinson’s Disease in Taiwan

**DOI:** 10.1371/journal.pone.0107465

**Published:** 2014-09-15

**Authors:** Yi-Jen Guo, Yi-Chu Liao, Ching-Heng Lin, Ming-Hong Chang

**Affiliations:** 1 Section of Neurology, Taichung Veterans General Hospital, Taichung, Taiwan; 2 Graduate Institute of Clinical Medical Science, China Medical University, Taichung, Taiwan; 3 Section of Neurology, Taipei Veterans General Hospital, Taipei, Taiwan; 4 Department of Neurology, National Yang-Ming University School of Medicine, Taipei, Taiwan; 5 Department of Medical education and Research, Taichung Veterans General Hospital, Taichung, Taiwan; University of Toronto, Canada

## Abstract

**Introduction:**

Several treatment guidelines for Parkinson’s disease (PD) had been proposed in recent decades. The aim of current study was to investigate the initial medication utilized in newly diagnosed PD subjects in Taiwan during an eleven-year period.

**Methods:**

A total of 7,550 patients with newly diagnosed Parkinsonism were retrospectively enrolled from the National Health Insurance Research Database of Taiwan from 2000 to 2010. After excluding patients at risk of secondary or atypical Parkinsonism, those never receiving medication or having incomplete data, 1,645 subjects were included. The participants were then divided into four treating regimen groups, namely levodopa (LD) only group, dopamine agonist (DA) only group, LD+DA group, and No-LD, No-DA group. The demographic data and medication retention rate were compared across the four treatment groups.

**Results:**

LD only and No-LD, No-DA regimens were the main initial choice of PD treatment in Taiwan. LD containing drugs were more often prescribed to the elderly population than the other two treatment regimens, while No-LD, No-DA medication was the major initial choice for younger patients. DA only regimen occupied only 3–4% of the initial PD prescriptions and was given predominantly by neurologists. Over the eleven-year period, there is a trend for the middle-aged population to receive medication containing LD as initial treatment. The one year retention rate of anti-Parkinsonism medication was around 30–50% in our population. Age, polypharmacy, change of one-year daily levodopa equivalent dosage and newly onset of dementia, stroke and psychiatric diseases all affect drug compliance in PD patients.

**Conclusions:**

This is the first long-term study to explore initial pharmacotherapies in an Asian PD population. We hope to provide evidence for adjusting government policies and public education of physicians and PD patients in the future.

## Introduction

Parkinson’s disease (PD) is the second most common neurodegenerative disease with the cardinal motor features of bradykinesia, resting tremor, rigidity and postural instability [1]. The incidence and prevalence of PD increase with age. In the United States, the estimated annual incidence and prevalence of PD are 12.3 per 100,000 and 106.9 per 100,000 [2], [3].

Current therapies for PD patients include levodopa (LD), dopamine agonist (DA), monoamine oxidase B inhibitor (MAO-B inhibitor), catechol-O-methyltransferase inhibitor (COMT inhibitor), anticholinergic drugs and amantadine [4]–[6]. After 4–6 years of treatment with LD, 40% of the patients developed motor fluctuations and another 40% had dyskinesia [7]. The incidence of motor complications was even higher in the younger PD population receiving LD treatment [7], [8]. In one series, more than 90% of young PD patients receiving therapies containing LD developed motor complications within 5 years [9]. As compared to LD, DAs had a lower risk of motor complications but more neuropsychiatric and systemic adverse effects. Therefore, the usage of DAs as an initial therapeutic choice was limited in the elderly patients [10]–[12]. There were many guidelines proposed for the management of PD over the past two decades [5], [6], [13]–. Several of them suggested using a MAO-B inhibitor in patients with mild disability, DAs in younger patients with moderate to severe disability and LD in elderly patients with prominent functional impairment [5], [15], [16], [18]. The annual total net ingredient cost for anti-Parkinsonism medication increased gradually from £37 million in 1998 to £130 million in 2010 in England since the introduction of non-ergot DAs and MAO-B inhibitors [19]. However, there have only been a limited number of studies discussing the trend of initial therapeutic choice for PD patients in the real world [20], [21].

The National Health Insurance (NHI) program was implemented in Taiwan since March 1995. It offers a comprehensive, unified and universal health insurance program to all citizens [22]. The aim of current study is to explore the initial choice of pharmacotherapies in newly diagnosed PD patients from 2000 to 2010 by using the National Health Insurance Research Database (NHIRD).

## Methods

### Data source

Data was obtained from Taiwan’s NHIRD, which is maintained by the National Health Research Institutes (NHRI) and overseen by the state-run Bureau of NHI (BNHI) for research purposes. The NHI program in Taiwan started in 1995 and covered over 99.5% of the population by the end of 2009 [23].

All research had been approved by Institutional Review Board of Taichung Veterans General Hospital (CE13262). No informed consents from participants were obtained because the data were analyzed anonymously. We used data from 2000 to 2010 for the current study. According to the NHIRD, random sampling of the cohort is achieved by using the linear congruential random number generation function of the Sun WorkShop Compiler C 5.0. The distributions of the random samples were representative of the Taiwanese population [22].

### Study population

Patients were defined as having Parkinsonism if they received an ICD-9-CM code of 332.x more than three times in the outpatient services or more than once during hospitalization between January 2000 and December 2010. Patients were excluded if (1) they were exposed to medication with a high risk of drug-induced Parkinsonism for more than three months before PD diagnosis (Table S1 in File S1) [24], (2) they had diseases potentially causing secondary or atypical Parkinsonism (Table S2 in File S1) [25], (3) they had never received any anti-Parkinsonism medication, or (4) if their data was incomplete. The remaining patients were included in the present study.

### Initial pharmacotherapy

**Figure 1:**
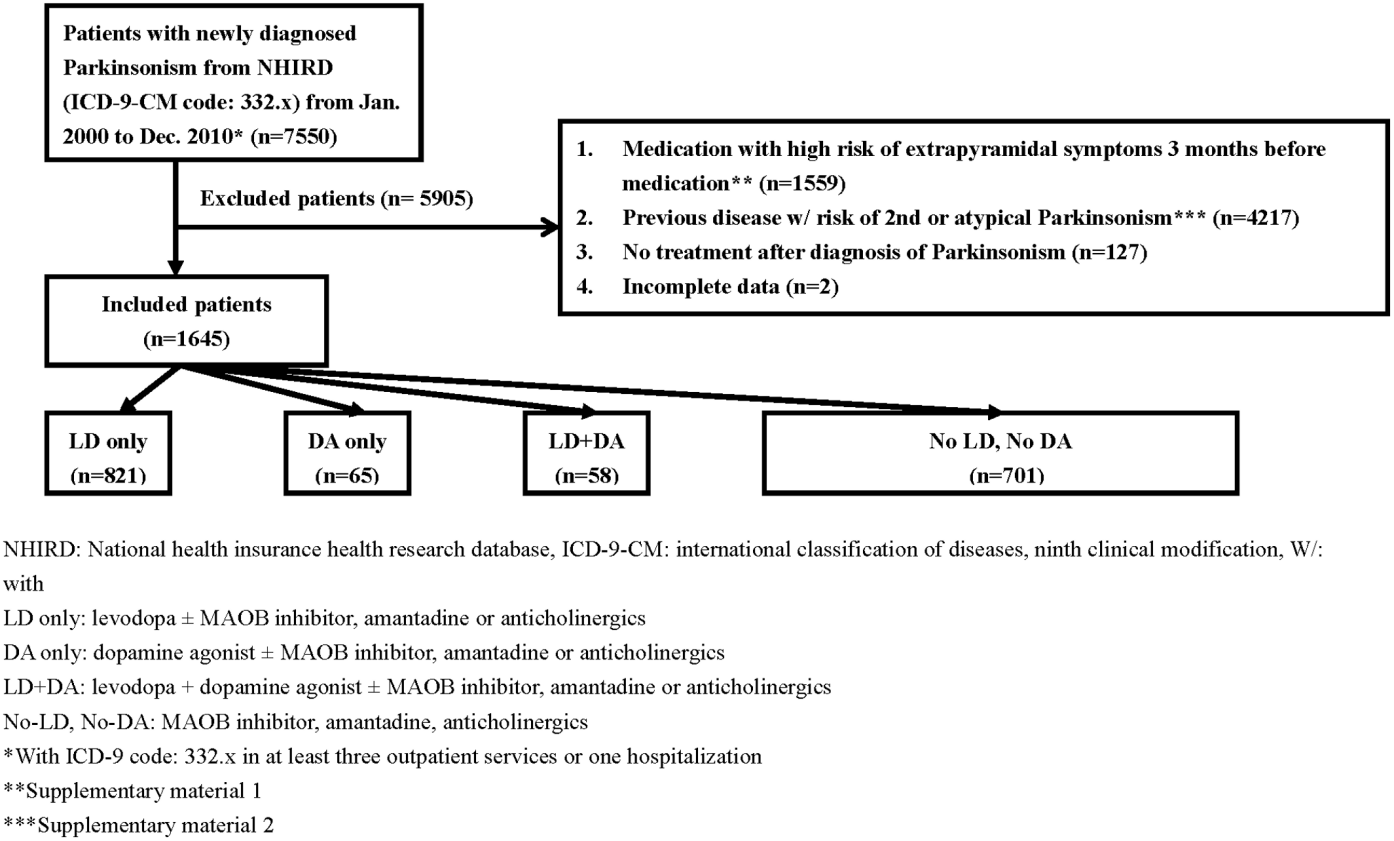
Flow chart of selection of PD patients.

The study participants were divided into four different treatment groups according to the initial medication used within the first year of PD diagnosis. The definitions of the four treatment groups were: (1) LD only group - Patients that received LD only or LD plus other anti-Parkinsonism medication other than DAs; (2) DA only group - Patients who took DAs only or DAs plus other anti-Parkinsonism drugs other than LD; (3) LD+DA group - Subjects who received a combination of LD and DAs regardless with or without other anti-Parkinsonism drugs; and (4) No-LD, No-DA group - Individuals who were given MAO-B inhibitors, anticholinergic drugs or amantadine as initial treatments (Fig. 1). Levodopa containing medication includes LD only and LD+DA drugs. All the anti-Parkinsonism drugs available in Taiwan are listed in Table S3 in File S1.

### Variables investigated

Data including age, gender, medical institution, year and doctors’ specialty of initial prescriptions, interval between PD diagnosis and prescription, initial and one-year daily levodopa equivalent dosage (LDED), initial drug cost and one year retention rate of anti-Parkinsonism medication were analyzed. Factors affecting the drug compliance of PD patients were also explored. One-year retention rate was defined as patients received medications confined to each treatment group for more than 6 months divided by the total number of patients in the same medication group. Any subject taking extra medication belonging to another treatment regimen group for more than three times was defined as a non-retention patient. For example, a subject in the LD only group was considered to be “non-retention patient” if DAs were prescribed for more than 3 times in subsequent follow-ups.

The definition of medication possession ratio (MPR) was the number of days with medication supply divided by 365 days (i.e. the follow-up period in this study). Having an MPR of more than 80% was classified as good compliance [26].

### Statistical analysis

Data was expressed as mean ± standard deviation or number (%). Categorical variables were compared by chi-squared test and continuous variables were compared by one-way analysis of variance (ANOVA). For analyses with significant p value in ANOVA, Scheffe’s test was performed for post-hoc analysis to evaluate the difference between groups. For categorical variables, chi-squared test was used for pairwise comparison between either two of the four treatment groups. All analyses were performed using SAS version 9.2 (SAS Institute, Cary, NC, USA). A p value of <0.05 was considered to be statistically significant.

## Results

A total of 7,550 subjects were diagnosed as having Parkinsonism in the NHIRD, and 1,645 patients fulfilled the inclusion criteria of newly diagnosed PD in the present study. They were assigned into four different regimen groups, including 821 patients in LD only group, 65 persons in DA only group, 58 persons in LD+DA group and 701 persons in No-LD, No-DA group (Fig. 1). The demographic data of the participants is shown in Table 1.

**Table 1:** Demographic characteristics of newly diagnosed PD patients, 2000–2010.

	First medication choice					
Variable	LD only	DA only	LD+DA	No-LD, No-DA	Total	P-value
	(N = 821)	(N = 65)	(N = 58)	(N = 701)	(N = 1645)	
Age, years old (mean ± SD)	70.2±11.0	65.0±13.3	71.0±11.4	56.7±18.8	64.3±16.3	<.0001^adefg^
<64, N (%)	214 (26.1)	27 (41.5)*	15 (25.9)	393 (56.1)*	649 (39.5)	<.0001
≥65, N (%)	607 (73.9)	38 (58.5)*	43 (74.1)	308 (43.9)*	996 (60.5)	
Gender						
Female, N (%)	400 (48.7)	28 (43.1)	30 (51.7)	364 (51.9)	822 (50.0)	0.41
Male, N (%)	421 (51.3)	37 (56.9)	28 (48.3)	337 (48.1)	823 (50.0)	
Medical institution						
Medical center, N (%)	267 (32.5)	28 (43.1)	23 (39.7)	200 (28.6)*	518 (31.5)	0.007
Regional hospital, N (%)	429 (52.3)	33 (50.7)	26 (44.8)	358 (51.0)*	846 (51.4)	
Clinic, N (%)#	125 (15.2)	4 (6.2)	9 (15.5)	143 (20.4)*	281 (17.1)	
Doctors’ specialty						
Neurologist, N (%)	481 (58.6)	53 (81.5)*	46 (79.3)*	211 (30.1)*	791 (48.1)	<.0001
Non-neurologist, N (%)	340 (41.4)	12 (18.5)*	12 (20.7)*	490 (69.9)*	854 (51.9)	
Year of prescription						
2000–2005, N (%)	431 (52.5)	31 (47.7)	38 (65.5)	419 (59.8)*	919 (55.9)	0.007
2006–2010, N (%)	390 (47.5)	34 (52.3)	20 (34.5)	282 (40.2)*	726 (44.1)	
Interval between PDdiagnosis and startingmedication (days)						
0, N (%)	686 (83.6)	44 (67.7)*	50 (86.2)	557 (79.5)	1337 (81.3)	0.0001
1–30, N (%)@	62 (7.6)	16 (24.6)*	2 (3.4)	53 (7.6)	133 (8.1)	
31–180, N (%)	22 (2.7)	2 (3.1)*	2 (3.4)	29 (4.1)	55 (3.3)	
≥181, N (%)	51 (6.2)	3 (4.6)*	4 (6.9)	62 (8.8)*	120 (7.3)	
Initial drug cost, NTD (mean ± SD)	248.3±270.5	605.2±778.1	641.1±641.1	238.7±895.9	272.2±652.7	<.0001^ahecg^
Initial daily Levodopaequivalent dosage^b^, mg/day(mean ± SD)	214.7±114.9	24.9±21.5	221.6±101.8	0	118.9±135.6	<.0001^adecf^
One-year daily Levodopaequivalent dosage^b^, mg/day(mean ± SD)	338.3±321.2	140.6±203.5	376.0±340.8	71.0±207.9	224.8±307.1	<.0001^adecf^
One year retention						
Non-retention case number, N (%)	452 (55.1)	44 (67.7)	29 (50.0)	416 (59.3)	941 (57.2)	0.07
Retention case number, N (%)	369 (44.9)	21 (32.3)	29 (50.0)	285 (40.7)	704 (42.8)	

a ANOVA test; chi-squared test for all other p-values.

b Only patients with medication from outpatient services were analyzed.

Post hoc test (Scheffe’s test) with statistical significance (p<0.05):

c LD only group differs from DA only group;

d LD+DA group differs from DA only group;

e LD+DA group differs from No-LD, No-DA group;

f LD only group differs from No-LD, No-DA group;

g DA only group differs from No-LD, No-DA group;

h LD+DA group differs from LD only group.

*p<0.05 compared to LD only group;

# p<0.05 compared to medical center group;

@p<0.05 compared to day 0 group.

PD: Parkinson’s disease, N: case number, %: percentage, SD: standard deviation, NTD: New Taiwan Dollar.

### Age as a major determinant of initial drug choice

Patients receiving No-LD, No-DA and DA only drugs as initial treatment were significantly younger in comparison to those taking LD only medication (56.7±18.8 y/o in No-LD, No-DA group, 65.0±13.3 y/o in DA only group, and 70.2±11.0 y/o in LD only group, Table 1). The trend was more obvious when the proportion of No-LD, No-DA patients were compared across different age groups (≦40, 41–64, and ≧65 y/o). For subjects younger than 40 y/o, up to 90.6% of PD patients received No-LD, No-DA drugs as initial treatment (Table 2). For the other two age groups, the percentage of No-LD, No-DA users dropped to 49.9% in middle age group and 30.9% in elderly age group respectively (all p values <0.0001).

**Table 2:** Initial medication choice in patients with newly diagnosed Parkinson’s disease according to age, 2000–2010.

Age, years old	Initial medication choice					P-value
	LD only	DA only*	LD+DA	No-LD, No-DA*	Total	
	(N = 821)	(N = 65)	(N = 58)	(N = 701)	(N = 1645)	
	N (%)	N (%)	N (%)	N (%)	N (%)	
≤40	12 (7.1)	4 (2.4)	0 (0.00)	154 (90.6)	170 (100.0)	<0.0001
41–64#	202 (42.2)	23 (4.8)	15 (3.1)	239 (49.9)	479 (100.0)	
≥65^#@^	607 (60.9)	38 (3.8)	43 (4.3)	308 (30.9)	996 (100.0)	

Chi-squared test for p-values.

* p<0.05 compared to LD only group;

# p<0.05 compared to age≤40 years old;

@p<0.05 compared to 41–64 years old group.

N: case number, %: percentage.

### Medical institutions and doctors’ specialty affected initial prescription

Most patients (51.4%) received their first anti-PD medication in regional hospitals (Table 1). Physicians in medical centers and regional hospitals had similar treating philosophy for PD (p = 0.219, df = 2). Majority of the DA only regimen was prescribed by doctors in the medical centers (43.1%) and in regional hospitals (50.7%), while only 6.2% of DA only regimen was prescribed by doctors in the clinics. 48.1% of PD patients received their initial prescription from neurologists. DAs only and DA+LD groups were mainly prescribed by neurologists than by other physicians (81.5% vs. 18.5% for DAs only group, and 79.3% vs.20.7% for DA+LD group respectively). The initial drugs choices for newly diagnosed PD were similar between neurologist in medical centers and those in regional hospitals (p = 0.805, df = 6, Table 3).

**Table 3:** Initial medication choice in patients with newly diagnosed Parkinson’s disease according to prescribing doctor, 2000–2010.

Prescribing doctors	Initial medication choice					P-value
	LD only	DA only*	LD+DA*	No-LD, No-DA*	Total	
	(N = 821)	(N = 65)	(N = 58)	(N = 701)	(N = 1645)	
	N (%)	N (%)	N (%)	N (%)	N (%)	
Neurologist of medical center	235 (62.0)	26 (6.9)	23 (6.1)	95 (25.1)	379 (100.0)	<0.0001
Non-neurologist of medical center#	32 (23.0)	2 (1.4)	0 (0.00)	105 (75.5)	139 (100.0)	
Neurologist of regional hospital	246 (59.7)	27 (6.6)	23 (5.6)	116 (28.2)	412 (100.0)	
Non-neurologist of regional hospital#	183 (42.2)	6 (1.4)	3 (0.7)	242 (55.8)	434 (100.0)	
Clinic#	125 (44.5)	4 (1.4)	9 (3.2)	143 (50.9)	281 (100.0)	

Chi-squared test for p-values;

*p<0.05 compared to LD only group;

# p<0.05 compared to neurologist of medical center group.

N: case number, %: percentage.

### Time trend of initial prescription and interval between PD diagnosis and medical intervention

**Figure 2:**
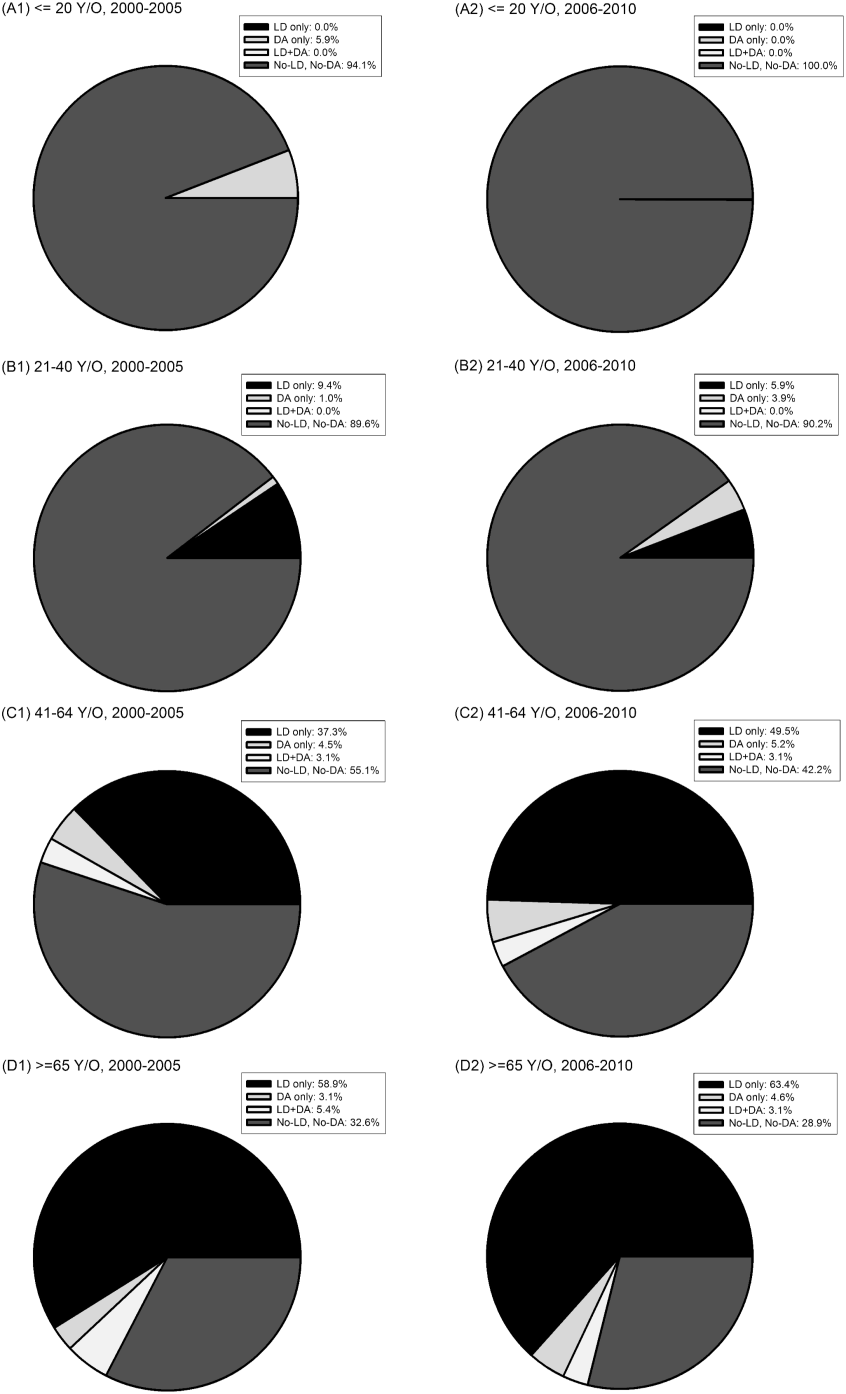
Trends of initial pharmacotherapies in different age groups between 2000–2005 and 2006–2010. (Compared by chi-squared test) (**A1**) ≤40 years-old, 2000–2005, (**A2**) ≤40 years-old, 2006–2010 (p = 0.643), (**B1**) 41–64 years-old, 2000–2005, (**B2**) 41–64 years-old, 2006–2010 (p = 0.042), (**C1**) ≥65 years-old, 2000–2005, (**C2**) ≥65 years-old, 2006–2010 (p = 0.099).

Patterns of initial pharmacotherapy choice changed between 2000–2005 and 2006–2010 (Table 1, p = 0.007). There was a trend for decreasing the proportion of No-LD, No-DA usage in passing decades (59.8% and 40.2% in 2000–2005 and 2006–2010 respectively). There were no significant differences between the percentage of drug choices before and after 2005 in other three treatment groups. In middle aged patients (41–64 y/o), the percentage of using LD only as initial medication increased from 37.3% to 49.5% before and after 2005. Besides, the percentage of No-LD, No-DA drugs decreased from 55.1% to 42.2% in the passing decade (p = 0.042, Fig. 2). The patterns of initial drug choice did not change between 2000–2005 and 2006–2010 for both neurologist and other physicians regardless what kind of medical institutions they worked for (Fig. S1).

Most PD medication (81.3%) was prescribed immediately after the patients were diagnosed with PD (Table 1). Among the four treatment groups, LD only regimen was more frequently chosen (51.3%). For subjects receiving DA only regimen, up to one-third (32.3%) had more than one month delay between the PD diagnosis and initiation of medical therapy (Table 1).

### Drug cost and dosage

Initial drug cost was significantly higher in DA only (605.2±778.1 NTD) and LD+DA groups (641.1±641.1 NTD) compared to LD only (248.3±270.5 NTD) and No-LD, No-DA groups (238.6±895.9 NTD) (p<0.05 in the post-hoc analysis). The initial daily LDED was statistically lower in DA only (24.9±21.5 mg/day) and No-LD, No-DA groups in comparison to the daily LDED in LD only (214.7±114.9 mg/day) and LD+DA groups (221.6±101.8 mg/day) (p<0.05 in the post-hoc analysis, Table 1) Similar trend was found in the one-year daily LDED analysis. (p<0.05 in the post-hoc analysis, Table 1).

### Drug compliance by one-year retention rate

The one year retention rate was around 30–50% in four treatment groups and there were no statistical differences among them (p = 0.07, Table 1). We then analyzed the retention rate of each treatment group after six to twelve months of initial treatment (Table 4). The most common reason for non-retention was medication withdrawal, and it accounted for 66.5% of the non-retention patients. 244 patients (25.9%) in the non-retention group needed medication containing LD (LD only or LD+DA medication) during the follow-up period (25.0% in LD only group, 34.1% in DA only group, 24.1% in LD+DA group and 26.2% in No-LD, No-DA group). No patient in the DA only group shifted back to a DA only regimen once non-retained.

**Table 4:** Medication of non-retention patients between 6 and 12 months after starting treatment.

Variable	Initial medicati					P-value
	LD only	DA only*	LD+DA*	No-LD, No-DA* ^#@^	Total	
	(N = 452)	(N = 44)	(N = 29)	(N = 416)	(N = 941)	
	N (%)	N (%)	N (%)	N (%)	N (%)	
LD only	4 (0.9)	8 (18.2)	6 (20.7)	91 (21.9)	109 (11.6)	<0.0001
DA only	9 (2.0)	0 (0.0)	2 (6.9)	11 (2.6)	22 (2.3)	
LD+DA	109 (24.1)	7 (15.9)	1 (3.4)	18 (4.3)	135 (14.3)	
No-DA, No-LD	38 (8.4)	6 (13.6)	4 (13.8)	1 (0.2)	49 (5.2)	
No medication	292 (64.6)	23 (52.3)	16 (55.2)	295 (70.9)	626 (66.5)	

Chi-squared test for p-values;

*p<0.05 compared to initial medication - LD only group;

# p<0.05 compared to initial medication - DA only group;

@p<0.05 compared to initial medication – LD+DA group; N: case number, %: percentage.

### Determinants of one-year retention rate – age, doctors’ specialty, medical institutions, one-year drug dosage and neuropsychiatric comorbidities

Patients younger than 65 years-old had a significantly higher retention rate as compared to those older than 65 years-old (46.7% and 40.3% respectively, p<0.05, Table 5).

**Table 5:** Clinical characteristics of retention and non-retention groups of patients with newly diagnosed Parkinson’s disease.

Variable	Retention group		Non-retention group		Total (N = 1645)	P-value
	(N = 704)		(N = 941)			
	MPR≥80%	MPR<80%	Keep medication	No-medication		
	(N = 233)	(N = 471)	(N = 315)	(N = 626)		
Age, years old (mean ± SD)	61.1±17.4	63.9±17.1	66.5±11.9	64.5±17.0	64.3±16.3	0.002^ac^
<64, N (%)	109 (46.8)	194 (41.2)	112 (35.6)*	234 (37.4)*	649 (39.5)	0.030
≥65, N (%)	124 (53.2)	277 (58.8)	203 (64.4)*	392 (62.6)*	996 (60.5)	
Gender						
Female, N (%)	134 (57.5)	230 (48.8)	146 (46.3)	312 (49.8)	822 (50.0)	0.066
Male, N (%)	99 (42.5)	241 (51.2)	169 (53.7)	314 (50.2)	823 (50.0)	
Initial medical institution						0.0002
Medical center, N (%)	65 (27.9)	173 (36.7)	119 (37.8)*	161 (25.7)	518 (31.5)	
Regional hospital, N (%)#	128 (54.9)	219 (46.5)	157 (49.8)*	342 (54.6)	846 (51.4)	
Clinics, N (%)#	40 (17.2)	79 (16.8)	39 (12.4)*	123 (19.6)	281 (17.1)	
Doctors’ specialty of initial prescription						
Neurologist, N (%)	90 (38.6)	235 (49.9)*	216 (68.6)*	250 (39.9)	791 (48.1)	<0.0001
Non-neurologist, N (%)	143 (61.4)	236 (50.1)*	99 (31.4)*	376 (60.1)	855 (51.9)	
Year of initial prescription						
2000–2005, N (%)	162 (69.5)	225 (47.8)*	164 (52.1)*	368 (58.8)*	919 (55.9)	<0.0001
2006–2010, N (%)	71 (30.5)	246 (52.2)*	151 (47.9)*	258 (41.2)*	726 (44.1)	
Major medical institution^f^						<0.0001
Medical center, N (%)	71 (30.5)	165 (35.0)	144 (45.7)*	152 (24.3)*	532 (32.3)	
Regional hospital, N (%)#	122 (52.4)	223 (47.3)	144 (45.7)*	289 (46.2)*	778 (47.3)	
Clinics, N (%)#@$	40 (17.2)	74 (15.7)	22 (7.0)*	117 (18.7)*	253 (15.4)	
Multiple hospitals, N (%)#@$	0 (0.0)	9 (1.9)	5 (1.6)*	68 (10.9)*	82 (5.0)	
Initial daily Levodopaequivalent dosage^g^, mg/day(mean ± SD)	108.8±130.7	134.5±139.8	110.9±116.8	114.8±143.1	118.9±135.6	0.039^a^
One-year daily Levodopaequivalent dosage^h^, mg/day(mean ± SD)	169.9±256.0	269.7±352.7	318.7±356.8	138.1±191.6	224.8±307.1	<0.0001^abcde^
Mortality, N (%)	56 (24.0)	98 (20.8)	74 (23.5)	167 (26.7)	395 (24.0)	0.162
Comorbidity^i^						
Stroke, N (%)	53 (22.7)	119 (25.3)	117 (37.1)*	148 (23.6)	437 (26.6)	<0.0001
Dementia, N (%)	33 (14.2)	73 (15.5)	72 (22.9)*	90 (14.4)	268 (16.3)	0.006
CNS trauma, N (%)	27 (11.6)	49 (10.4)	53 (16.8)	76 (12.1)	205 (12.5)	0.056
Sepsis, N (%)	32 (13.7)	48 (10.2)	48 (15.2)	77 (12.3)	205 (12.5)	0.186
Congestive heart failure, N (%)	19 (8.2)	32 (6.8)	30 (9.5)	68 (10.9)	149 (9.1)	0.126
Liver decompensation, N (%)	7 (3.0)	11 (2.3)	13 (4.1)	18 (2.9)	49 (3.0)	0.545
Renal failure, N (%)	15 (6.4)	24 (5.1)	15 (4.8)	47 (7.5)	101 (6.1)	0.262
Respiratory failure, N (%)	30 (12.9)	42 (8.9)	35 (11.1)	90 (14.4)	197 (12.0)	0.046
Neoplasm, N (%)	19 (8.2)	31 (6.6)	23 (7.3)	59 (9.4)	132 (8.0)	0.359
Psychiatric disorders, N (%)	108 (46.4)	178 (37.8)*	160 (50.8)*	214 (34.2)	660 (40.1)	<0.0001

N: case number, %: percentage, MPR: medical possession rate, No medication: receive no anti-PD medication during the 7^th^ to 12^th^ month after starting medication, CNS: central nervous system.

a ANOVA test; chi-squared test for all other p-values.

Post hoc test (Scheffe’s test) with statistical significance (p<0.05):

b Retention (MPR≥80%) group differs from Retention (MPR<80%) group;

c Retention (MPR≥80%) group differs from Non-retention (Keep medication) group;

d Retention (MPR<80%) group differs from Non-retention (No-medication) group;

e Non-retention (Keep medication) group differs from Non-retention (No-medication) group.

*p<0.05 compared to MPR≥80% group;

# p<0.05 compared to medical center group;

@p<0.05 compared to regional hospital group;

$ p<0.05 compared to clinics group.

f The medical institution from where patients received ≥50% medication during the first year. Subjects would be classified into multiple hospitals group if they received medication <50% from each level of medical institution.

g Only patients with medication from outpatient services were analyzed.

h The last prescription of patients with medication from outpatient services during one-year follow-up were analyzed.

i Definition of comorbidities: patients with diseases of the following ICD codes for more than three times during the outpatient services or once during hospitalization within one year after the diagnosis of Parkinson’s disease. Stroke: 430–438/A290-A294, A299; Dementia: 290, 331.0, 331.2/A210; CNS trauma: 344, 800, 801, 803, 804, 805, 806, 850, 851, 852, 853, 854, 959.01/A470, A490, A491, 952; Sepsis: 038, 020.0, 790.7, 117.9, 112.5, 112.81; Congestive heart failure: 398.91, 402.01, 402.11, 402.91, 404.01, 404.03, 404.11, 404.13, 404.91, 404.93, 425.4–425.9, 428; Liver decompensation: 570, 571.2, 571.5, 571.6, 572.2, 572.4, 567.0, 567.2, 567.8, 567.9, 789.5, 456.0, 456.1, 456.2; Renal decompensation (renal failure): 584, 585, 586, V451, V56; Respiratory failure: 5188; Neoplasm: 140–208 (excluding 195–199); Psychiatric disorders: 290–313.

Using patients with MPR≥80% as a reference, more PD patients who received initial treatment from neurologists and medical centers would shift to other drugs during the one year follow-up period (p<0.05). The ratio of shifting medication was 23.0% in medical centers, 18.6% in regional hospitals and 19.1% in clinics. 27.3% of patients receiving initial medication from neurologists would shift medication during the study period while 11.6% of subjects with initial drugs from non-neurologists would change their drugs. Patients taking medication from multiple medical institutions had the highest ratio of no medication after one year of starting treatment in comparison to those receiving major treatment (≥50% of medication) from the same level of medical institutions (82.9% in multiple hospitals, 46.2% in clinics, 37.1% in regional hospitals and 28.6% in medical centers, p<0.05).

Pot-hoc analysis showed no significant differences of initial daily LDED in retention and non-retention patients. Patients in the groups of MPR<80% and shift-medication had a higher one-year daily LDED in comparison to subject with MPR≥80% and those without medication (318.7±356.8 mg/day in shift medication group, 269.7±352.7 mg/day in MPR<80% group, 169.9±256.0 mg/day in MPR≥80% group and 138.1±191.6 mg/day in no medication group, p<0.05).

Mortality and most systemic morbidities, such as CNS trauma, sepsis, congestive heart failure, liver decompensation, renal failure and neoplasms, didn’t affect drug compliance of PD patients. However, patients in the shift-medication group had a significantly higher percentage of newly onset of stroke (37.1%), dementia (22.9%) and psychiatric diseases (50.8%) when compared to those with MPR≥80%.

## Discussion

This is the first long-term study to explore the initial pharmacotherapies in an Asian PD population. The differences with regards to treatment strategies for newly diagnosed PD patients were compared between Taiwan PD patients and the proposed guidelines for Western populations. Factors affecting initial drug choice and medical compliance were analyzed.

The initial drug choices preference for newly diagnosed young PD patients was different between Taiwan and Western countries. Doctors in Taiwan preferred to give No-LD, No-DA medication for younger patients (60.6%), and the ratio was significantly higher than the percentage in Western populations (7–33%) [20], [21]. About 15–20% of young PD patients (<65 y/o) in Western countries used DAs as their initial treatment and the percentage was much higher than that in the present study (4.2%). There are a number of possible explanations for this phenomenon. First, the BNHI has strict budget limitations for doctors which could predispose physicians to choose more cost-effective medications. Second, seeking medical service was more convenient and cheaper for Taiwanese patients when compared to patients in the United States. Patients would be more likely to change their doctors if their initial medication was less potent or caused more adverse effects. Third, the demographic features of study participants were different between current study and the related literature reports. The percentage of patients younger than 65 years-old (39.5%) was higher in the present research as compared to the studies of Kari Swarztrauber et al. and Daniel M. Huse et al. (15–20%) [20], [21]. The ratio of participants treated by neurologists in our study was more than 1.5 times when compared to the study by Kari Swarztrauber et al. Patterns of initial pharmacotherapies in PD patients changed in Taiwan during the passing eleven years. The major difference included decreasing usage of No-LD, No-DA regimen and increasing LD usage as initial PD regimen in patients of 41–64 years-old. Patients of this age group are the major economic support of their family. As a result, they might need more effective drugs for symptomatic control. To balance the patient needs and budget limitations, LD containing regimen is the preferred regimen for most doctors in Taiwan.

Compared to Western PD populations, the one year retention rate was lower (30–50%) in Taiwan. However, the determinants of medication compliance were similar between Western PD patients and subjects in Taiwan [26], [28]–[30]. In the related literature, the medication possession ratio in newly treated PD patients was around 50–75% during the first year [26], [28]. In the present study, only 32.5% of patients could maintain medication uninterrupted during their first year [26]. Subjects with older ages, initial prescribing year between 2006 and 2010, receiving their first drugs from non-neurologists, receiving drugs from institutions other than medical centers, polypharmacy, higher one-year daily LDED and neuropsychiatric comorbidities such as stroke, dementia and psychiatric disorders had suboptimal medical persistence. Gender, mortality rate and other systemic comorbidities didn’t influence medical possession condition of PD population in Taiwan. Participants who received medication from medical centers and neurologists more frequently changed their medication during follow-up. The better availability of different regimens in these medical institutions might be one of the explanations for this finding. 25.9% of non-retention patients changed their medication to a LD containing regimen. The percentage of early PD patients who needed to add LD in LD-naïve therapy within two years was 17.2% in the REAL-PET study and 53% in the CALM-PD study [10], [27]. The medication withdraw rate was up to 38.1% among all patients. Although we established a strict exclusion criteria, some patients with secondary or atypical Parkinsonism such as drug-related Parkinsonism or dementia with Lewy bodies might still be included in our analysis [24], [25]. Some doctors may use levodopa in the beginning to observe the clinical response for diagnostic purposes. These would also contribute to the low retention rate in this study.

There were a number of limitations that should be highlighted with regards to our studies. First, we could not obtain the medical charts from the NHIRD. Therefore, we could not completely exclude the possibility to include secondary Parkinsonism or atypical Parkinsonism in our study participants. We acknowledge this would affect the results of one-year retention rate because patients with dementia of Lewy bodies have a poorer drug response and higher risk of encountering neuropsychiatric adverse effects. However, doctors might still treat patients who had only Parkinsonism symptoms and didn’t show overt cognitive decline as PD patients in the beginning. Second, we were not sure about the functional status of Taiwanese PD patients who started seeking medical help which would influence initial medical choice and subsequent drug titration. We analyzed the medications of these subjects after one year of starting treatment which might reflect the truly clinical status and suitable medication for these patients. Third, we didn’t include all participants in NHIRD for analysis. However, a prior study showed that the distributions of the sampling results were representative of the Taiwanese population [22].

## Conclusion

This is the first retrospective research to discuss the initial medication in newly diagnosed PD patients in an Asian population and the possible explanations for divergence of treatment of our patients from guidelines proposed by specialists. Although, further reports from other countries are needed to establish a clear picture about PD treatment in the real world, we hope to provide evidence to encourage the adjustment of government policies and public education of physicians and PD patients in the future.

## Supporting Information

Figure S1 **Trends of initial pharmacotherapies with doctors’ specialty and medical institutions during the eleven years.** (Compared by chi-squared test) **(A1–2)** Neurologists from centers (p = 0.127), **(B1–2)** Non-neurologists from centers (p = 0.637), **(C1–2)** Neurologists from regional hospitals (p = 0.319), **(D1–2)** Non-neurologists from regional hospitals (p = 0.963), **(E1–2)** Clinics (p = 0.182).
(TIF)

File S1 **Table S1 in File S1.** Drugs with high risk of extrapyramidal symptoms. **Table S2 in File S1.** Diseases with risk of of 2nd or atypical Parkinsonism. **Table S3 in File S1.** Available medication for treating Parkinson’s disease in Taiwan between 2000–2010.
(DOCX)
