# Supplementary figures and images for: Initial Medication in Patients of Newly Diagnosed Parkinson’s Disease in Taiwan
Source: PLoS One. 2014 Sep 15;9(9):e107465. doi: 10.1371/journal.pone.0107465 (PMC4164642; doi:10.1371/journal.pone.0107465)

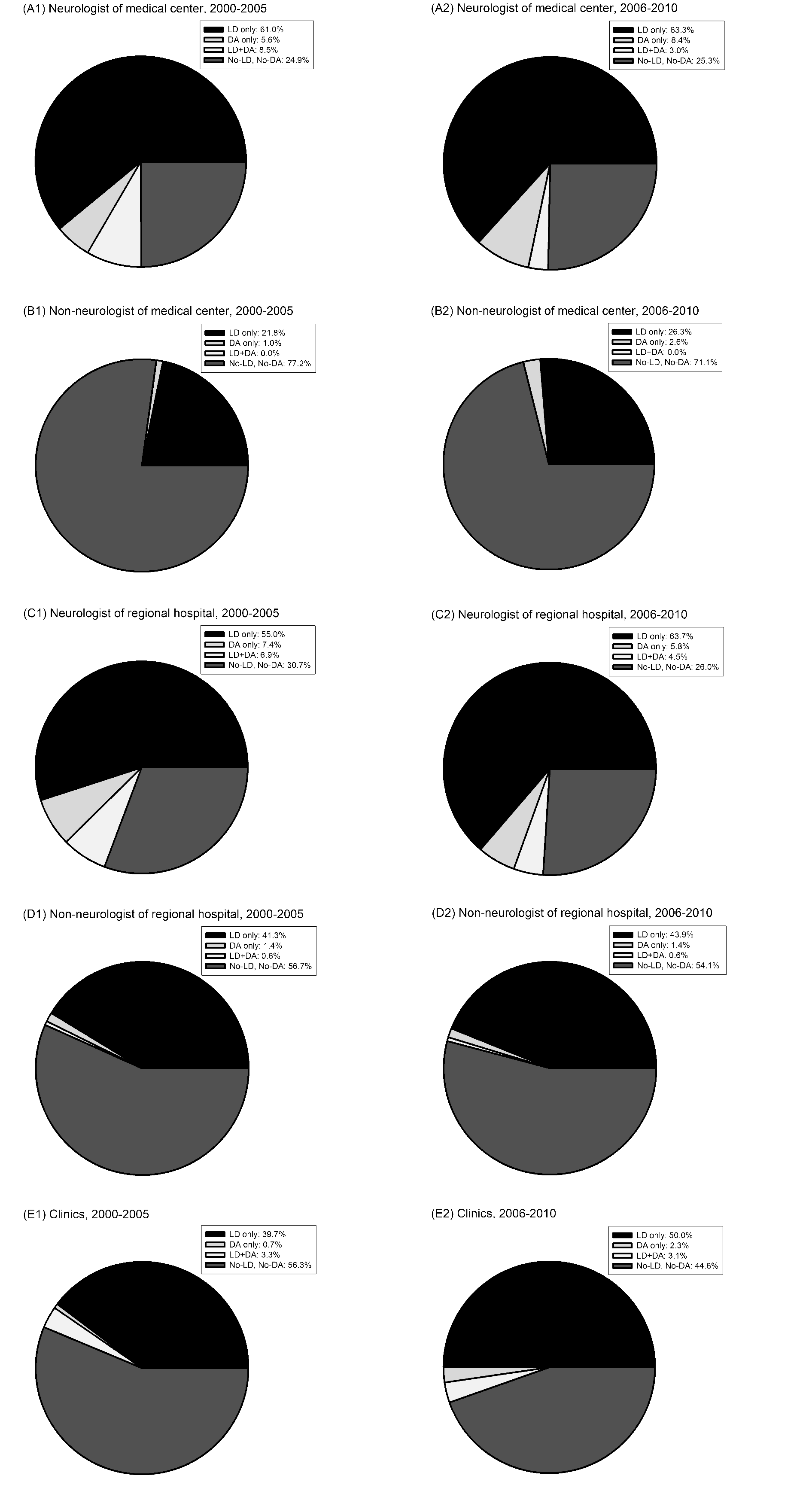

Supplement: Figure S1 — Trends of initial pharmacotherapies with doctors’ specialty and medical institutions during the eleven years. (Compared by chi-squared test) (A1–2) Neurologists from centers (p = 0.127), (B1–2) Non-neurologists from centers (p = 0.637), (C1–2) Neurologists from regional hospitals (p = 0.319), (D1–2) Non-neurologists from regional hospitals (p = 0.963), (E1–2) Clinics (p = 0.182). (TIF) [file pone.0107465.s001.tif]
