# Supplementary material for: Initial Medication in Patients of Newly Diagnosed Parkinson’s Disease in Taiwan
Source: PLoS One. 2014 Sep 15;9(9):e107465. doi: 10.1371/journal.pone.0107465 (PMC4164642; doi:10.1371/journal.pone.0107465)
Supplement: File S1 — Table S1 in File S1. Drugs with high risk of extrapyramidal symptoms. Table S2 in File S1. Diseases with risk of of 2nd or atypical Parkinsonism. Table S3 in File S1. Available medication for treating Parkinson’s disease in Taiwan between 2000–2010. (DOCX) [file pone.0107465.s002.docx]

**Table S1. Drugs with high risk of extrapyramidal symptoms.**

| Drug category | Generic drugs |
| --- | --- |
| Anti-emetics | Metoclopramide, Prochlorperazine |
| Neuroleptics | Haloperidol, Amisulpride, Flupentixol, Fluphenazine, Levomepromazine, Pimozide, Amisulpride, Thioridazine, Zuclopenthixol, Risperidone, Olanzapine, Aripiprazole |
| Calcium channel blocker | Flunarizine, Cinnarizine |
| Dopamine depleter | Reserpine, Tetrabenazine |
| Dopamine synthesis blocker | Methyldopa |

**Table S2. Diseases with risk of of 2^nd^ or atypical Parkinsonism.**

| Diseases | ICD-9 code |
| --- | --- |
| Stroke | 430-438/A290-A294,A299 |
| Dementia | 290,331.0, 331.2/A210 |
| Meningitis, encephalities | 00321,﻿0065,﻿0130,﻿0131,﻿0132,﻿0133,﻿0136,﻿0360,﻿0361,﻿0460,﻿0461,﻿0462,﻿0463,﻿047,﻿0490,﻿0491,0520,﻿0530,﻿0543,﻿05472,﻿0550,﻿05601,﻿062,﻿063,﻿064,﻿0721,﻿0722,﻿09041,﻿09042,﻿0941,﻿0942,﻿09481,﻿09482,﻿09487,﻿09882,﻿10081,﻿11283,﻿1142,﻿11501,﻿11511,﻿11591,﻿1300,﻿1390,﻿320,321,322,323,3240,3249,﻿325,326 |
| Head injury | 800, 801, 803, 804, 850, 851, 852, 853, 854 |
| Hydrocephalus | 742.3, 741.0, 331.3, 331.4 |
| Brain tumor | 191, 192.0, 192.1, 192.8, 192.9, 194.3, 194.4, 198.3, 237.0, 237.1, 237.5, 237.6, 237.9, 239.6, 239.7, 2250, 2252, ﻿2273, 2274, ﻿22802 |
| Congenital or hereditary disorders | 2750, 2751,﻿ 3334,﻿ 334, ﻿740 |
| Hypoxic encephalopathy | 348.1, 997.01, 639.8, 669.4, 768.7, 779.2 |

**Table S3. Available medication for treating Parkinson’s disease in Taiwan between 2000-2010.**

| Classes | Names | Introduction date |
| --- | --- | --- |
| Levodopa | Levodopa | 1 April 1997 |
|  | Levodopa+Benserazide | 1 March 1995 |
|  | Levodopa+Carbidopa | 1 March 1995 |
|  | Levodopa+Entacapone+Carbidopa | 1 September 2006 |
| COMT inhibitor | Entacapone | 1 July 2002 |
| Dopamine agonist | Pramipexole | 1 November 2002 |
|  | Ropinirole | 1 February 2002 |
|  | Bromocriptine | 1 March 1995 |
|  | Lisuride | 1 March 1995 |
|  | Pergolide | 1 March 1995 |
|  | Cabergoline | 1 June 2002 |
|  | Apomorphine | 1 December 2001 |
| MAO-B inhibitor | Selegiline | 1 March 1995 |
| Anti-cholinergic drugs | Trihexyphenidyl | 1 March 1995 |
|  | Biperiden | 1 March 1995 |
|  | Benztropine | 1 March 1995 |
|  | Orphenadrine | 1 March 1995 |
| Others | Amantadine | 1 March 1995 |
